# Formulation of Phytosomes with Extracts of Ginger Rhizomes and Rosehips with Improved Bioavailability, Antioxidant and Anti-Inflammatory Effects In Vivo

**DOI:** 10.3390/pharmaceutics15041066

**Published:** 2023-03-25

**Authors:** Mariana Deleanu, Laura Toma, Gabriela Maria Sanda, Teodora Barbălată, Loredan Ştefan Niculescu, Anca Volumnia Sima, Calin Deleanu, Liviu Săcărescu, Alexandru Suciu, Georgeta Alexandru, Iuliana Crişan, Mariana Popescu, Camelia Sorina Stancu

**Affiliations:** 1 Lipidomics Department, Institute of Cellular Biology and Pathology "Nicolae Simionescu" of the Romanian Academy, 8 B.P. Haşdeu Street, 050568 Bucharest, Romania; 2 “Costin D. Nenitescu” Institute of Organic and Supramolecular Chemistry of the Romanian Academy, 202B Splaiul Independenței Street, 060023 Bucharest, Romania; 3 “Petru Poni” Institute of Macromolecular Chemistry of the Romanian Academy, Aleea Grigore Ghica Voda 41A, 700487 Iasi, Romania; 4 Hofigal Export Import S.A., 2 Intrarea Serelor, 042124 Bucharest, Romania

**Keywords:** antioxidant, anti-inflammatory, bioavailability, phytosomes, ginger, rosehips

## Abstract

The poor water solubility of natural antioxidants restricts their bioavailability and therapeutic use. We aimed to develop a new phytosome formulation with active compounds from extracts of ginger (GINex) and rosehips (ROSAex) designed to increase their bioavailability, antioxidant and anti-inflammatory properties. The phytosomes (PHYTOGINROSA-PGR) were prepared from freeze-dried GINex, ROSAex and phosphatidylcholine (PC) in different mass ratios using the thin-layer hydration method. PGR was characterized for structure, size, zeta potential, and encapsulation efficiency. Results showed that PGR comprises several different populations of particles, their size increasing with ROSAex concentration, having a zeta potential of ~-21mV. The encapsulation efficiency of 6-gingerol and β-carotene was >80%. ^31^P NMR spectra showed that the shielding effect of the phosphorus atom in PC is proportional to the amount of ROSAex in PGR. PGR with a mass ratio GINex:ROSAex:PC-0.5:0.5:1 had the most effective antioxidant and anti-inflammatory effects in cultured human enterocytes. PGR-0.5:0.5:1 bioavailability and biodistribution were assessed in C57Bl/6J mice, and their antioxidant and anti-inflammatory effects were evaluated after administration by gavage to C57Bl/6J mice prior to LPS-induced systemic inflammation. Compared to extracts, PGR induced a 2.6-fold increase in 6-gingerol levels in plasma and over 40% in the liver and kidneys, in parallel with a 65% decrease in the stomach. PGR treatment of mice with systemic inflammation increased the sera antioxidant enzymes paraoxonase-1 and superoxide dismutase-2 and decreased the proinflammatory TNFα and IL-1β levels in the liver and small intestine. No toxicity was induced by PGR either in vitro or in vivo. In conclusion, the phytosome formulation of GINex and ROSAex we developed resulted in stable complexes for oral administration with increased bioavailability, antioxidant and anti-inflammatory potential of their active compounds.

## 1. Introduction

Various therapies have been developed to treat pathologies such as cardiovascular diseases (CVD), diabetes, hepato-steatosis, cancer, arthritis or inflammatory bowel disease that are induced or aggravated by increased oxidative and inflammatory stress [1,2]. Many of these therapies have failed completely or partially, while others, although successful, are accompanied by considerable side-effects (statins, chemotherapy drugs, nonsteroidal drugs) [3]. Some studies have reported benefits for antioxidants as complementary treatment, but no clear benefit of an antioxidant therapy has yet been demonstrated in CVD or cancer studies [4]. An explanation for this failure could be the high concentration of antioxidants used in these studies since, more recently, it has been observed that high doses of antioxidants can be rather harmful [5]. Another explanation is that some antioxidants become unstable in circulation. For example, polyphenols are compounds with a very high antioxidant potential, but their bioavailability is very limited due to their rapid enzymatic modification in vivo, which blocks the hydroxyl groups required for their antioxidant effect [4]. The importance of antioxidants is due not only to their free radical scavenging activity, but also their gene regulation properties, such as carotenes, polyphenols, α-tocopherol and flavonoids, which have been demonstrated to modulate many genes involved in the regulation of receptors, enzymes, transcription factors or proteins of various signaling cascades [4].

The recent trend to use natural compounds or medicinal plants for preventive purposes or associated with the recommended allopathic drug treatment for the pathologies mentioned above is increasing [3]. Some important aspects should be considered in preparing natural compounds for therapies: (i) the stability of the active compounds extracted from the plants and (ii) their bioavailability in order to have considerable beneficial effects relative to the administered dose with minimal or no side effects. Ginger (*Zingiber officinale*) rhizomes are used as spices in the preparation of foods and drinks and in traditional medicine as a remedy since ancient times [6,7]. Scientific evidence shows that ginger has antioxidant [8], anti-inflammatory [9], hypolipidemic [10,11], antidiabetic [12], immune modulatory, antitumor [13], antiapoptotic and anticoagulant properties [8,14]. In addition, ginger extracts impede the biofilm formation for many pathogenic bacteria and enhance the antifungal properties of medication such as fluconazole, which is very useful for curing diseases caused by drug-resistant pathogens [15]. The active compounds of ginger trigger multiple signaling pathways to exert their therapeutic effects as follows: (i) the regulation of cellular metabolic processes is performed by interfering in the adenosine-monophosphate-dependent protein kinase, insulin-like growth factor 1 and insulin signaling pathways; (ii) antiapoptotic effects are based on the regulation of cell proliferation, cell survival and differentiation through the serine/threonine-specific protein kinase pathways; (iii) antitumoral properties are due to the regulation of the epidermal growth factor receptor pathway; and (iv) the promotion of fatty acid catabolism is performed by stimulating the peroxisome proliferator activated receptor γ pathway [7]. More than 400 different compounds have been identified in ginger extracts, the most abundant active principles being gingerols and shogaols [7]. The most abundant type of gingerol in fresh ginger rhizomes is 6-gingerol, followed by 10-gingerol and 8-gingerol; the dehydration of gingerols generates the corresponding shogaols [16]. The low bioavailability of orally ingested gingerols is due to their low solubility. A couple of negative side effects have been described for ginger including digestive tract irritation, cardiac arrhythmias and bleeding due to the interaction with anticoagulants; most of these effects occurred due to the high doses used with the intent to increase the benefits [17]. The methods used to enhance gingerols’ solubility and bioavailability and to prevent harmful interactions comprise formulations of gingerols as microemulsions or nanoparticles. Some liposomal ginger formulations were developed in order to increase their bioavailability, but their beneficial effects remain to be demonstrated by in vivo studies [18]. There are only a few papers reporting the anti-inflammatory or neuroprotective effects of phytosomes containing extracts of ginger and mulberry or ginger and *Acmella oleracea* [19,20].

The fruits of rosehip (*Rosa canina*) have long been used in traditional medicine worldwide for the treatment of arthritis, to reduce pain or to decrease glucose and cholesterol plasma levels. Over one hundred compounds have been identified in rosehips, the major active components being lipophilic, such as flavonoids, anthocyanins, phenolic compounds, fatty oils and organic acids. Scientific studies evidenced numerous pharmacological activities for rosehip including antioxidant, anti-inflammatory, anti-obesity, anti-cancer, hepatoprotective, nephroprotective, cardioprotective, neuroprotective and anti-rheumatic [21,22]. These active compounds have different targets in the nuclear factor kappa B signaling pathway, such as inhibiting proinflammatory enzymes (e.g. matrix metalloproteinases), lowering the production of inflammatory cytokines and chemokines (tumor necrosis factor α—TNFα, interleukin-1β and -6—IL-1β, IL-6) and reducing the oxidative stress, which in turn alleviates inflammatory processes [23]. For many years, the powder or the aqueous extracts of rosehips have been used to prove their beneficial effects [23]. In the last decade, the hydroalcoholic extracts of rosehips have been tested for their beneficial effects exerted in beta cells and proved their antidiabetic properties [24]. No formulations of the active compounds of rosehip extracts in phytosomes have been reported until now.

There are many commercially available natural products in which the manufacturers claim to contain ginger or rosehip active principles individually, but they are formulated as powder capsules or alcoholic extracts that have poor stability allowing for consistent losses in their concentration in vivo, diminishing the compounds’ bioavailability; thus, the administration of high doses becomes necessary to obtain the beneficial effects [25]. High doses of ginger extract are associated with adverse effects [17]. There are also formulations of liposomes carrying different natural active compounds for therapeutic use [26]. Important physicochemical differences between new generation phytosomes and liposomes have been described which result in important advantages for the first: (i) phytosomes are more stable given the H-bonds formed between phospholipids and the carried active compounds, whereas no chemical bonds are formed in liposomes and (ii) the molar ratio of natural compounds and phospholipids is 1:1 or 1:2 in phytosomes, which confers a very low susceptibility to oxidation, while in liposomes, the percentage of oxidation-prone phospholipids surrounding the water-soluble compounds is high [25].

In the present study, we aimed to establish a method to prepare phytosomes with bioactive compounds from hydroalcoholic extracts of ginger rhizomes and rosehip fruits designed to increase their bioavailability, biodistribution and antioxidant and anti-inflammatory properties.

## 2. Materials and Methods

### 2.1. Reagents

All used reagents were from Sigma-Aldrich Co., Saint Louis, MO, USA. Soy phosphatidylcholine was from Avanti Polar Lipids, Inc. (Alabaster, Alabama, USA). For Western blot (WB) analysis, the following antibodies were used: mouse anti-superoxide dismutase 2 (SOD2) (1:400, sc-1331340), mouse anti-TNFα (1:400, sc-52746), mouse anti-IL-1β (1:400, sc-52012), mouse anti-β-actin (1:4000, sc-47778) and rabbit anti-mouse IgG H&L (HRP) (1:10000, ab6728), which were from Santa Cruz Biotechnology, Dallas, TX, USA and Abcam, Cambridge, UK. The foetal calf serum (FCS) was from Euroclone, Pero, Milan, Italy, EU, the 100 kDa cutoff Amicon centrifugal filter columns were from Millipore (Billerica, MA, USA), Trizol was from Ambion, (ThermoFisher Scientific, Waltham, MA, USA), primers were from Biolegio BV (Nijmegen, Netherland) and M-MLV Reverse Transcriptase and SyBr Select Master Mix were from Invitrogen (ThermoFisher Scientific, Waltham, MA, USA). The HPLC-grade solvents were from Merck (Kenilworth, NJ, USA).

### 2.2. Preparation and Characterization of Ginger Extracts

The protocol and the methods used for the preparation and characterization of the hydroalcoholic ginger rhizome extract (GINex) were previously described [10,11,27]. Briefly, fresh ginger rhizomes (*Zingiber officinale Roscoe*) were sliced, oven-dried at 60 °C and ground as fine powder that was subjected to extraction with 80% ethanol (*v*/*v*) in an ultrasonic bath (60 min, 65 °C), followed by centrifugation (3500× *g*, 15 min) and evaporation of ethanol from the supernatant in a rotary evaporator (40 °C). The GINex was freeze dried at −100 °C for ~5 days using a Scanvac CoolSafe Touch 100-9 system (Labogene) and stored at −20 °C until use.

The freeze-dried GINex was characterized in terms of total polyphenols (TP), total flavonoids (TF), 6-gingerol, 6-shogaol and total gingerol content. The amount of TP from GINex was determined by the Folin–Ciocâlteu method with some modifications [10]. The TF content was determined according to the Dowd method [10]. To assess the gingerol composition of GINex, a chromatographic separation of the compounds was performed as previously described [10,11,27] using ultrahigh performance liquid chromatography (UHPLC) equipped with a diode array detector (DAD) (Agilent Technologies 1290 Infinity). All these methods are detailed in the Supplementary Materials.

### 2.3. Preparation and Characterization of Rosehip Extract

Whole rosehip fruits were sectioned, dried at 70 °C for 48 h and ground into powder and were subjected to extraction with 90% ethanol *(v/v*) at 50 °C under magnetic stirring for 6 h. The resulting mixture was filtered and concentrated at a reduced pressure, at 40 °C until the alcohol evaporated. The rosehip extract (ROSAex) was freeze dried as described above and stored at −20 °C until use. The freeze-dried ROSAex was characterized in terms of carotenoids, TP and TF. The total carotenoid content was determined by a previously described UHPLC method [28] which is detailed in the Supplementary Materials. The contents of TP and TF in ROSAex were determined as described above for GINex.

### 2.4. Preparation of Phytosomes

The phytosomes termed PHYTOGINROSA (PGR) and PHYTOGIN (PG) were prepared by the thin-layer hydration method [29]. Briefly, accurately weighted quantities of GINex and ROSAex (Table 1) were placed in a round bottom flask and dissolved in 100 mL of absolute ethanol at 60 °C under reflux and magnetic stirring until a homogeneous suspension was obtained. Separately, soy phosphatidylcholine (PC) was solubilized in 25 mL of absolute ethanol and slowly added under reflux and stirring to the solubilized GINex and ROSAex that reacted for 2 h at 60 °C. Ethanol was then evaporated under a low pressure in a rotary evaporator. The resulting sticky/oily, translucent, orange-colored compound was dried under nitrogen stream to remove any traces of the solvent. The dried PGR and PG were hydrated with water (80 mL), and the particle size was adjusted by sonication in a water bath for 30 min. The phytosomes were characterized in terms of size, polydispersity, zeta potential, encapsulation efficiency, drug loading and surface morphology and were freeze dried at −100 °C for ~5 days using a Scanvac CoolSafe Touch 100-9 system (Labogene) for long-term preservation.

### 2.5. Characterization of PGR and PG Phytosomes

The analysis of physical characteristics (size, size distribution, zeta potential), encapsulation efficiency and morphology by transmission electron microscopy (TEM) was conducted on the phytosomes reconstituted in ultrapure water at a ratio of 1:10 (phytosomes:water), followed by sonication for 30 min in a water bath.

#### 2.5.1. Size and Zeta Potential

The average particle size, polydispersity index (PDI) and zeta potential of the phytosomes were determined by dynamic light scattering (DLS) and electrophoretic light scattering (ELS) techniques, respectively, using a Malvern Zetasizer Nano-ZS (ZEN3600, Malvern Instrument Ltd., Malvern, UK) equipped with a 633 nm laser. The samples were diluted from 1 µL to 2 mL with ultrapure water so that we had an optimal scattering intensity. Scattered light intensities at an angle of 173° were measured using a standard operating method [30]. For each sample, the reported size average of the intensity distribution represents the mean of three runs. In the same measuring cuvette, after determining the size, an electrode Universal Dip Cell (ZEN1002) was immersed into the sample to determine the zeta potential by using ELS. We performed 5 consecutive measurements at 5 V with a delay of 300 sec between measurements. Results were analyzed using the built-in ZetaSizer Software 7.12 (Malvern Instruments).

#### 2.5.2. Surface Morphology

The surface morphology was studied using the TEM technique. The procedure involved the placing of a sample drop of reconstituted phytosomes in water (1:10) on a copper grid covered with carbon for 1 min. The excess solution was removed using a filter paper; then, the sample was investigated using an HT7700 transmission electron microscope (Hitachi) operating at 100 kV in high contrast mode.

#### 2.5.3. Encapsulation Efficiency and Drug Loading

To determine the encapsulation efficiency of the phytosomal formulation, the non-entrapped free 6-gingerol and β-carotene were separated from the phytosomal emulsion by ultracentrifugation using an Amicon Ultra 100 kDa filter membrane at 14.000×g for 30 min. Then, the concentrations of 6-gingerol and β-carotene were measured in the filtrate by UHPLC according to the methods described above. 

The encapsulation efficiency (EE, %) and drug loading (%) were calculated using the following formulas:

$$\mathrm{EE}\;\left(\%\right)=\frac{\left(\mathrm{initial}\;\mathrm{amount}\;\mathrm{of}\;6\;\mathrm{gingerol}\right)-\left(\mathrm{amount}\;\mathrm{of}\;\mathrm{free}\;6\;\mathrm{gingerol}\;\mathrm{from}\;\mathrm{filtrate}\right)}{\mathrm{initial}\;\mathrm{amount}\;\mathrm{of}\;6\;\mathrm{gingerol}}\times 100$$

$$\mathrm{Drug}\;\mathrm{loading}\;\left(\%\right)=\frac{\left(\mathrm{initial}\;\mathrm{amount}\;\mathrm{of}\;6\;\mathrm{gingerol}\right)-\left(\mathrm{amount}\;\mathrm{of}\;\mathrm{free}\;6\;\mathrm{gingerol}\;\mathrm{from}\;\mathrm{filtrate}\right)}{\mathrm{initial}\;\mathrm{amount}\;\mathrm{of}\;\mathrm{phytosomes}}\times 100$$

We proceeded similarly to determine the EE (%) and the drug loading of β-carotene.

#### 2.5.4. In Vitro Bioaccesibility Study

A standardized in vitro digestion method was used to investigate the bioaccessibility of 6-gingerol from extracts + PC (physical mixture) versus PGR 0.5:0.5:1. The samples, accurately weighted, were incubated in conditions that sequentially simulated gastric (SGF) and small intestinal (SIF) fluids in accordance with the INFOGEST protocol [31] with minor modifications. Briefly, 10 mL of SGF for digestion was prepared from 7.5 mL of SGF electrolytes (INFOGEST), 2 mL of pepsin (31.2 mg/mL), 0.295 mL of water and 5 µL of CaCl_2_ (0.3 M), and the pH was adjusted to 2 using 3 M HCl. Then, 3 mL of SGF digesta was added to the samples and incubated at 37 ° C for 2 h with continuous shaking at 100 rpm/min. After 2 h, 3 mL of SIF digesta (1:1) was added to the mixture. The solution was prepared from 11 mL of SIF electrolytes (INFOGEST), 5 mL of pancreatin (8 mg/mL), 3.61 mL of water and 40 µL of CaCl_2_ (0.3M), and the pH was adjusted to 7.0 using 1 M NaOH (total volume 20 mL). The samples were incubated at 37 °C for 2–3 h with continuous shaking at 100 rpm/min. At the end of the in vitro digestion, the digested sample was used to measure the bioaccessibility percentage. The digested samples were vigorously vortexed and centrifuged at 13,500× *g* for 30 min at 4 °C. The supernatant was collected and filtered on a 0.22 µm membrane filter. The 6-gingerol content was quantified by the UHPLC method described above, and the bioaccessibility was calculated as follows:

$$\mathrm{Bioaccessibility}\;\left(\%\right)=\frac{\mathrm{Cdigesta}}{\mathrm{Ci}}\times 100$$

where

Cdigesta = amount of 6-gingerol from simulated gastrointestinal fluids. 

Ci = initial amount of 6-gingerol in extracts and PGR 0.5:0.5:1

#### 2.5.5. Physical Storage Stability Test

A long-term stability test of the freeze-dried PGR 0.5:0.5:1 was evaluated immediately after preparation and subsequently at regular time intervals, according to Wanjiru et al. [32]. Drug residual loading (%) was determined by UHPLC in the PGR compared to GINex stored at 4 °C at 0, 1, 3, 6, and 12 months.

#### 2.5.6. Structure Determination by Nuclear Magnetic Resonance (NMR) Spectroscopy

The NMR technique was used to structurally characterize the PGR. NMR spectra were recorded in deuterated chloroform (CDCl_3_) in 5 mm Wilmad 507 tubes at a concentration of 50 mg/mL of PC, PG and PGR on a Bruker Avance Neo 400 spectrometer. ^31^P NMR spectra were recorded at 161.97 MHz over a 400 ppm spectral window with 128 scans, a 0.5 sec acquisition time, a 2 sec relaxation time, a 30° pulse, and WALTZ16 hydrogen decoupling.

### 2.6. In Vitro Evaluation of PGR

#### 2.6.1. Cell Culture

Epithelial cells from human colon (Caco-2 cell line, ATCC, Manassas, VA, USA) were used to test the cytotoxic effects of PGR compared to the extracts. Caco-2 cells seeded at a density of 200.000/mL were grown according to the manufacturer’s instructions in minimum essential media (MEM) supplemented with FCS (10%, *v/v*), non-essential amino acid solution (1%), penicillin (100 U/mL), streptomycin (0.1 mg/mL) and l-glutamine (4 mM).

#### 2.6.2. Selection of the Optimal Formulation and Concentration of PGR Depending on the Antioxidant and Anti-Inflammatory Properties

Confluent Caco-2 cells were preincubated for 6 h with the culture medium containing PGR; then, the inflammatory molecule TNFα (20 ng/mL) was added to the medium for another 18 h. TNFα was used to induce oxidative and inflammatory stress in cells [27]. The cells were incubated with PGR in the three formulations: GINex:ROSAex:PC 0.9:0.1:1, 0.75:0.25:1 and 0.5:0.5:1. Four concentrations of each formulation expressed as equivalent 6-gingerol were tested: 5, 10, 20 and 40 µM. In parallel, we used control cells incubated only with TNFα (TNFα) or cells incubated with TNFα and PC (PC) in a concentration equivalent to that of PGR. After 24 h of incubation, the cells were processed for the gene expression of some indicator proteins of the antioxidant (paraoxonase 2—PON2, SOD2) and inflammatory (IL-1β) status.

##### Quantitative Real-Time PCR

To measure the gene expression, total RNA from cells was isolated using the TRIzol reagent. In total, 1–2 μg of the obtained RNA was reverse-transcribed using the enzyme MultiScribe Reverse Transcriptase according to the manufacturer’s recommendations. The obtained complementary DNA was amplified using SyBr Select Master Mix and gene-specific primers for SOD2, PON2, IL-1β and β-actin as housekeeping gene (Table S1). For amplification, the ViiA7 Real-Time PCR system was used (Applied Biosystems, Foster City, CA, USA). Relative quantification of the amplification products was conducted comparative to cells incubated with TNFα alone to which we assigned a value of 1.

#### 2.6.3. Evaluation of the PGR 0.5:0.5:1 Cytotoxicity 

Confluent Caco-2 cells were incubated with PGR 0.5:0.5:1 or the corresponding concentration of the mix GINex + ROSAex at 20 µM of equivalent 6-gingerol. In parallel, cells incubated with PC or ethanol in similar concentrations with those present in PGR and hydroalcoholic extracts were used as controls.

##### Estimation of Cells’ Viability

The cell viability was estimated using XTT (Sigma Aldrich Co., Saint Louis, MO, USA) and the CytoTox-ONE™ Homogeneous Membrane Integrity Assay (Promega, Madison, WI, USA). In brief, at the end of the incubation period, a mixture of XTT (2,3-bis-(2-methoxy-4-nitro-5-culfophenyl)-2H-tetrazolium-5-carboxanilide) and phenazine methosulfate (PMS) was added to each well. After 4h at 37 °C, the absorbance of the formed formazan was detected with the Tecan Infinite M200 Microplate Reader (Tecan, Austria) at 450 nm. The CytoTox-ONE™ Homogeneous Membrane Integrity Assay is an enzymatic method that measures the intracellular lactate dehydrogenase (LDH). After incubation, the cells were lysed, and the protocol of the manufacturer was respected. The fluorescence of the final product of the reaction was measured at 560 nm/590 nm.

##### Measurement of the Energetic Status of the Cells

The energetic status of the cells was estimated by measuring the intracellular ATP levels with a commercially available kit (ViaLight Assay, Lonza, Rockland, ME, USA), according to the manufacturer’s instructions.

### 2.7. In Vivo Evaluation of PGR 0.5:0.5:1

#### 2.7.1. Animal Model

A lot of 30 C57Bl/6J 15-week-old male mice with 25.45 ± 1.39 gram body weights (stock No: 000664, The Jackson Laboratory) were used. The mice were housed in the specific pathogen-free (SPF) animal facility of the Institute of Cellular Biology and Pathology ”Nicolae Simionescu” and were maintained at a 24 °C constant ambient temperature with 12 h/12 h alternating cycles of dark/light. All experimental procedures were carried out in accordance with the EU Directive 2010/63/EU on the protection of animals used for scientific purposes, were approved by the Ethics Committee of the Institute of Cellular Biology and Pathology “Nicolae Simionescu” (Approval no. 10/09.10.2019) and received the authorization no. 593/20.01.2020 from the National Sanitary Veterinary and Food Safety Authority.

##### Measurement of the Free 6-Gingerol Levels in the Plasma and Organs of Mice

Bioavailability of free 6-gingerol in the plasma

A lot of C57Bl/6J mice received a maximum volume of 200 µL of PGR 0.5:0.5:1 by gavage reconstituted in water or the equivalent mix of GINex and ROSAex in a single dose of 9.2 mg 6-gingerol/kg body weight. After 15, 30, 45, 60 and 120 min, the animals were anesthetized using a ketamine/xylazine cocktail (50–150 mg/kg ketamine and 5–10 mg/kg xylazine, i.p. injection), and blood was collected by cardiac puncture in EDTA-containing tubes. The resulting blood samples (n = 3 animals/time point) were kept on ice, and the plasma obtained by centrifugation at 2000× *g* was stored at –80 °C until the evaluation of the free 6-gingerol levels was conducted using a method adapted from [33] as described below at the Section “Samples preparation for measurement of free 6-gingerol”.

Biodistribution of free 6-gingerol in organs

At 60 and 120 min post-administration of PGR 0.5:0.5:1 or extract mix on the model presented above (Bioavailability of free 6-gingerol in plasma), the anesthetized animals were subjected to laparotomy, and their vasculatures were perfused with phosphate saline buffer (PBS) to remove the blood; then, their organs (stomach, liver, kidney, spleen, lung, pancreas, heart) were collected for the measurement of free 6-gingerol. The collected organs were cut into small pieces and frozen in liquid nitrogen. The tissue samples were then stored at −80 °C until processing.

Sample preparation for the measurement of free 6-gingerol

Accurately weighted tissue samples (<200 mg) were homogenized in 200 µL of saline buffer on ice. After this, 10 µL of internal standard (IS) nonivamide 5 µg/mL in acetonitrile and 790 µL of acetonitrile or 390 µL of acetonitrile for 100 µL of plasma were added to each sample (homogenized tissue or plasma) [33]. The samples were vigorously vortexed and centrifuged at 13,500× *g* for 15 min. The supernatant was evaporated in nitrogen stream. The residue was reconstituted in 100 μL of 80% ethanol, vortexed for 1 min and centrifuged at 13,500× *g* for 10 min at room temperature. In total, 10 µL of the supernatant was injected into the UHPLC Agilent Technologies 1290 Infinity instrument equipped with DAD and a 4-channel binary pump.

UHPLC determination of free 6-gingerol from plasma and organs

Chromatographic separation was performed on a Zorbax SB-C18 column (2.1 × 100 mm, 1.8 µm, Agilent Technologies, Santa Clara, CA, USA) with a column temperature of 50 °C. The mobile phase was composed by 0.1% formic acid solvent (A) and 0.1% formic acid acetonitrile solvent (B). The separation of the compounds was carried out with a gradient elution profile: 0 min—40% B, 1 min—40% B, 9 min—80% B, 10 min—95% B, 13 min—95% B, 15 min—40% B and 16 min—40% B with a 0.3 mL/min flow rate. The detection was conducted at λ = 282 nm. The standard curve of 6-gingerol was constructed by analyzing solutions with different concentrations (100, 200, 400, 800, 1600, 3200 ng/mL) using IS nonivamide (500 ng). The correlation coefficient (R^2^ = 0.99938) showed that the assay method was linear and acceptable for quantitative analysis. The injection volume was 10 µL. The results were expressed as free 6-gingerol ng/g tissue and ng/mL plasma, respectively. Agilent ChemStation software was used for data acquisition and processing.

Assessment of the antioxidant and anti-inflammatory effects of PGR 0.5:0.5:1

To evaluate the antioxidant and anti-inflammatory effects of PGR 0.5:0.5:1 in vivo, a lot of C57BL/6J mice were randomly divided into 3 groups that received by gavage 100 µL of (1) PGR 0.5:0.5:1 containing 4.5 mg of 6-gingerol/kg body weight (PGR, n = 8), (2) a mix of extracts GINex and ROSAex containing the same concentration of 6-gingerol/kg body weight as in PGR 0.5:0.5:1 (Ex, n = 8) or (3) PC in the same concentration as in PGR 0.5:0.5:1 (PC, n = 7). A group of animals was maintained as a control (no treatment) (C, n = 7). 

We chose the dose of PGR based on a pilot study to establish the dose for bioavailability/biodistribution experiments and based on the data existing in the literature. In the bioavailability/biodistribution study, we established that a dose of 9.2 mg/kg body weight of 6-gingerol is adequate to detect the level of active compound in plasma. Based on our results and those of the literature that mentioned a therapeutic dose of 5 mg/kg body weight administrated to mice [6], we chose 4.5 mg/kg body weight as the working dose that had no side effects.

After 7 days, the animals from the three groups of treatment received a sublethal dose of lipopolysaccharides (LPS) (0.2 mg LPS/kg body weight) by intra-peritoneal administration to provoke systemic inflammation as described [34]. After 16–18 h of free access to standard chow and water, the mice were anesthetized using an overdose of ketamine/xylazine cocktail, and ~500 µL of blood was collected by cardiac puncture and was kept for 30 min at room temperature and centrifuged at 10.000×g for 2 min to obtain serum. Serum samples were kept at −80 °C until the evaluation of antioxidant enzymes PON1 (protein levels and enzymatic activity), SOD2 protein, the pro-oxidant enzyme myeloperoxidase (MPO) enzymatic activity and the level of proinflammatory TNFα. The anesthetized animals were subjected to laparotomy and their vasculatures were perfused with PBS; then, their livers and small intestines were collected for the measurement of proinflammatory markers TNFα and IL-1β. The collected organs were cut into small pieces and frozen in liquid nitrogen. The tissue samples were then stored at -80 °C until processing.

Measurement of PON1 enzymatic activity in serum

PON-1 activity was measured as its capacity to hydrolyze paraoxon substrate using a method previously described [35].

Measurement of TNFα levels in serum

TNF-α levels in the serum of mice were measured using the commercially available kit ELISA Mouse TNF-α DuoSet (R&D Systems, Minneapolis, MN, USA) according to the manufacturer’s instructions.

Measurement of MPO enzymatic activity in serum

MPO activity was measured using an MPO Peroxidation Assay Kit (700160, Cayman Chemical, Ann Arbor, MI, USA) according to the manufacturer’s instructions.

Western blotting analysis

The tissue samples of the livers and small intestines from the control mice and animals treated with PGR, Ex and PC were homogenized in radioimmunoprecipitation assay buffer (RIPA) on ice and were then centrifuged for 10 min at 10,000× *g* at 4 ° C. The supernatants were collected and assessed for the total protein concentration using the bicinchoninic acid method. Equal amounts of tissue protein (50 μg) were loaded on 10–12% sodium dodecyl-sulfate polyacrylamide gel (SDS-PAGE) and processed for WB analysis of TNFα, IL-1β and β-actin as a reference protein using specific antibodies mentioned in the Reagents section.

Evaluation of the PGR 0.5:0.5:1 toxicity in vivo

To evaluate the toxicity of PGR 0.5:0.5:1 in vivo, we measured the levels of alanine aminotransferase (ALT) activity and creatinine in the plasma of the mice used in the biodistribution experiment at 60 and 120 min and in the sera of mice with LPS-induced systemic inflammation.

Measurement of ALT activity

The ALT activity, as an indicator of hepatic toxicity [36], was measured in the plasma and sera of mice after PGR 0.5:0.5:1 or extract administration. A commercially available kit based on the measurement of NADH oxidation was used according to the manufacturer’s instructions (Dialab, Neudorf, Austria).

Measurement of creatinine level

The creatinine levels were measured in the plasma and sera of mice after PGR 0.5:0.5:1 or extract administration as an indicator of renal dysfunction [37]. A commercially available kit (Dialab, Neudorf, Austria) based on the Jaffé method was used according to the manufacturer’s instructions.

### 2.8. Statistical Analysis

Statistical analysis and graphical representations were performed using the GraphPad 9.0 software (GraphPad Software Inc., San Diego, CA, USA) and SPSS for Windows 26 (IBM SPSS, IBM Ireland, Dublin, Ireland). Differences between the continuous variables such as the parameters measured in the cell cultures, plasma and tissues of mice were evaluated using the independent Student’s T-test for the comparison between the study groups. The trend of the variation in the parameters measured following in vitro experiments was calculated using the one-way ANOVA test followed by the Bonferroni post hoc test. Data were expressed as means ± SD, and *p* < 0.05 was set as the threshold for the statistical significance.

## 3. Results

### 3.1. Characterization of GINex and ROSAex

Ginger rhizomes were oven dried at 60 °C followed by extraction with 80% ethanol. We chose this mild temperature in order to obtain the highest antioxidant capacity for ginger in accordance with previously reported data [38,39]. The yield extraction after freeze drying was high (33.8%). The content of TP was 41% (Table 2), higher than that reported by Mustafa and Chin [40]. The obtained GINex was 24 times richer in TP compared to TF. The 6-gingerol content represents 3% of the freeze-dried extract and 7.5% of TP, respectively. The 6-shogaol content in GINex, which is usually generated by 6-gingerol dehydration during processing or storage, was 20 times lower than that of 6-gingerol. The profile of gingerols and shogaols are presented as corresponding chromatograms in Figure S1.

It is generally known that rosehips are rich in bioactive compounds such as carotenoids, and previous reports showed that the total carotenoids in rosehips are in the range of 8–49 mg/100 grams dried weight [41]. Whole rosehip fruits were sectioned, dried at 70 °C for 48 h and ground and were subjected to extraction. Our goal was to obtain an extract (ROSAex) with a high content of carotenoids. For this purpose, we tested several concentrations of ethyl alcohol, and the 90% concentration proved to be the best one in order to obtain the highest carotenoid content in the extract. The yield extraction after the freeze drying of ROSAex was 23 ± 3%. The β-carotene content of ROSAex was 0.297 ± 0.092 grams/100 grams, which represents ~86% of the total carotenoids determined by the UHPLC method. The profile of the carotenoids is presented as the corresponding chromatograms in Figure S2. The TP content determined by the Folin–Ciocalteu method was 5.42 ± 0.09 grams/100 grams, eight times lower than the TP of GINex (Table 3).

### 3.2. Characterization of PGR

#### 3.2.1. Size and Zeta Potential of PGR

**Figure 1:**
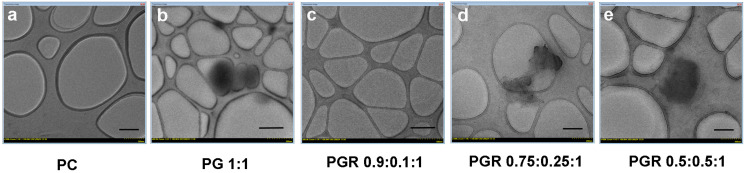
Transmission electron micrographs showing the morphology of the three types of PHYTOGINROSA (PGR) formulations compared to phosphatidylcholine (PC) and PHYTOGIN (PG); (**a**,**d**,**e**)—scale bar 200 nm, (**b**,**c**)—scale bar 500 nm.

The average hydrodynamic diameters of the phytosome particles are ~190 nm for PGR 0.9:0.1:1, ~330 nm for PGR 0.75:0.25:1 and ~780 nm for PGR 0.5:0.5:1 (Table 4). The PGR 0.5:0.5:1 particles have a high polydispersity index of 0.5 as a result of the presence of several populations. The phytosomes are negatively charged between -18 and −22 mV, which indicates a relatively good stability (Table 4). The generally accepted value of −30 mV is required for a stable system [42]. The successful preparation of stable phytosomes with low zeta potential values was previously reported [43]. PGR formulations have a high polydispersity index due to the presence of several different populations of particles; their size increases as the concentration of ROSAex increases, in agreement with the TEM micrographs shown in Figure 1.

#### 3.2.2. Surface Morphology

The TEM micrographs show that the PC particles are spherical and polydisperse, and those of PG are elongated with a high degree of polydispersity. In the case of PGR, the particles grow larger as the percentage of the ROSAex content increases as presented in Figure 1, in agreement with the size measurements (Table 4). The PGR 0.5:0.5:1 particles present an aggregation tendency.

#### 3.2.3. Encapsulation Efficiency and Drug Loading

The main bioactive compounds of GINex and ROSAex are 6-gingerol and β-carotene. We have evaluated their encapsulation in phytosomes and quantified their concentration by the UHPLC method described above. The concentrations of 6-gingerol and β-carotene in each type of PGR are presented in Table 5. It can be observed that both 6-gingerol and β-carotene have a high encapsulation efficiency between 83.3% and 94.4%. The loading of 6-gingerol and β-carotene in phytosomes was determined by UHPLC, and the corresponding chromatograms are presented in Figures S1 and S2.

#### 3.2.4. In Vitro Bioaccesibility

**Figure 2:**
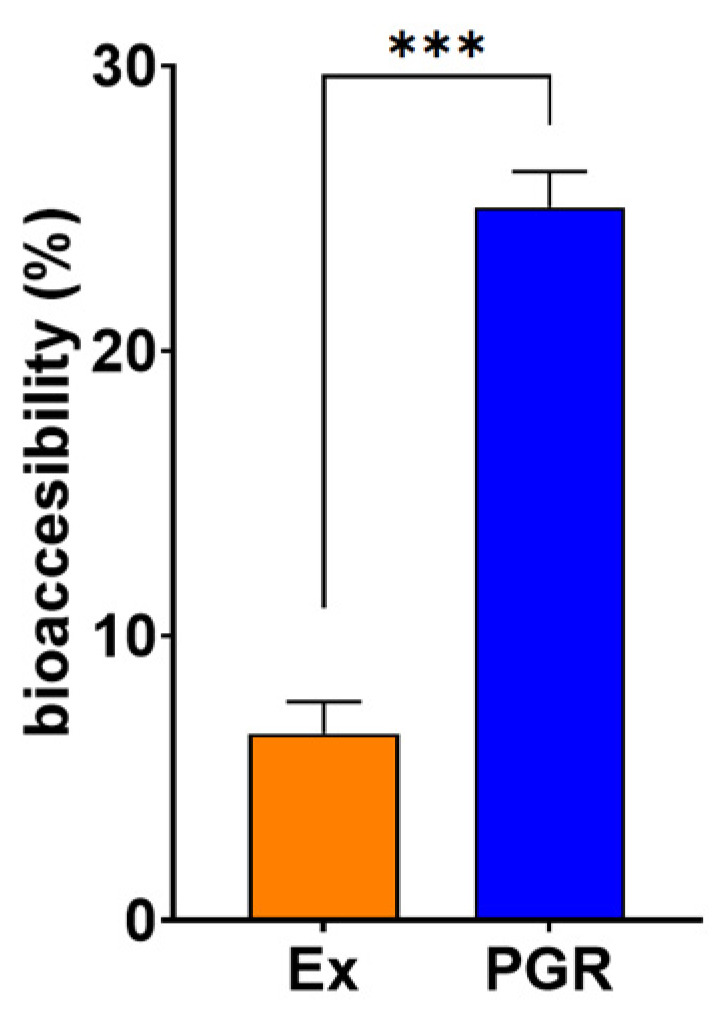
The bioaccessibility of PHYTOGINROSA (PGR) 0.5:0.5:1 compared to that of extracts (Ex) after exposure to simulated gastrointestinal conditions. The results are presented as means ± SD. *** *p* < 0.001 for PGR vs. Ex.

Bioaccessibility can be defined as the quantity of a compound available for absorption after gastrointestinal (GI) digestion, whereas bioavailability refers to the extent to which a compound enters the systemic circulation to exert bioactivity. The GI tract plays a key role in modulating the bioaccessibility and subsequent bioavailability of polyphenols in vivo [44]. The bioaccesibility of PGR 0.5:0.5:1 was four times higher than that of the respective extracts, indicating a much better stability and solubility of PGR than those of the extracts (Figure 2). The extensive degradation and the very low solubility of the extracts were due to the conditions (digestive enzymes and the acidic environment) that mimic those of the GI tract. These findings are in good agreement with previous reports showing the low bioaccessibility of free phenolic compounds after in vitro digestion as compared to encapsulated polyphenols [32,45].

#### 3.2.5. Physical Storage Stability Test

The drug-loading residual content is an essential indicator for the long-term stability of freeze-dried PGR 0.5:0.5:1 and was evaluated at predetermined time intervals (0, 1, 3, 6 and 12 months). Results evidenced that the 6-gingerol concentration in PGR 0.5:0.5:1 remained constant for one year at 4°C, while the concentration of 6-gingerol in GINex decreased by 33% under the same conditions (Table 6). An explanation for the preservation of the 6-gingerol content is the phytosomal formulation that allows for the formation of hydrogen bonds between the polar heads of PC and polyphenols and, in addition, the presence of carotenoids from ROSAex, which are powerful antioxidants.

#### 3.2.6. Structure Determination by Nuclear Magnetic Resonance (NMR) Spectroscopy 

**Figure 3:**
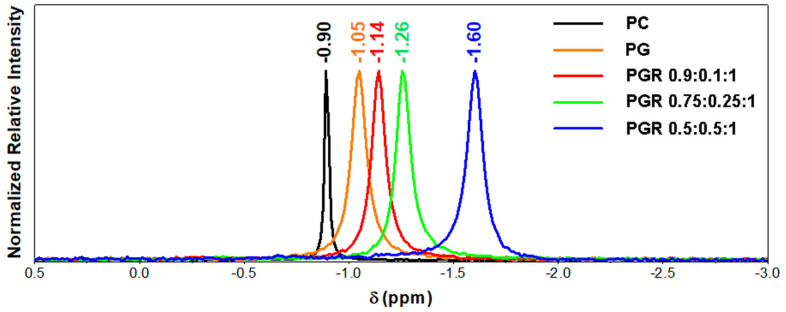
The ^31^P NMR spectra of PHYTOGINROSA (PGR) compared to PHYTOGIN (PG) and phosphatidylcoline (PC).

The ^31^P NMR spectrum of PC has a signal at the chemical shift of −0.90 ppm. The shielding effect is proportional to the amount of ROSAex content in the PGR. Thus, the chemical shift is −1.05 in PG and −1.14, −1.26 and −1.60 ppm for PGR 0.9:0.1:1, PGR 0.75:0.25:1 and PGR 0.5:0.5:1, respectively. In the ^31^P NMR spectra, the signal of ^31^P in CDCl_3_ is at −1.06 ppm and is 35.3 Hz wide, which corresponds to complexed phospholipids in accordance with European Patent EP 1 837 030 A1 [46]. PGR at a concentration of 5% *w/v* is soluble in CDCl_3_, and the NMR spectra confirm the formation of the complex. In PG, only GINex is complexed, and the chemical shift of −1.05 is observed. The overlaid spectra recorded for each preparation are shown in Figure 3.

### 3.3. Selection of the Optimal Formulation and Concentration of PGR Depending on the Antioxidant and Anti-Inflammatory Properties Determined In Vitro

**Figure 4:**
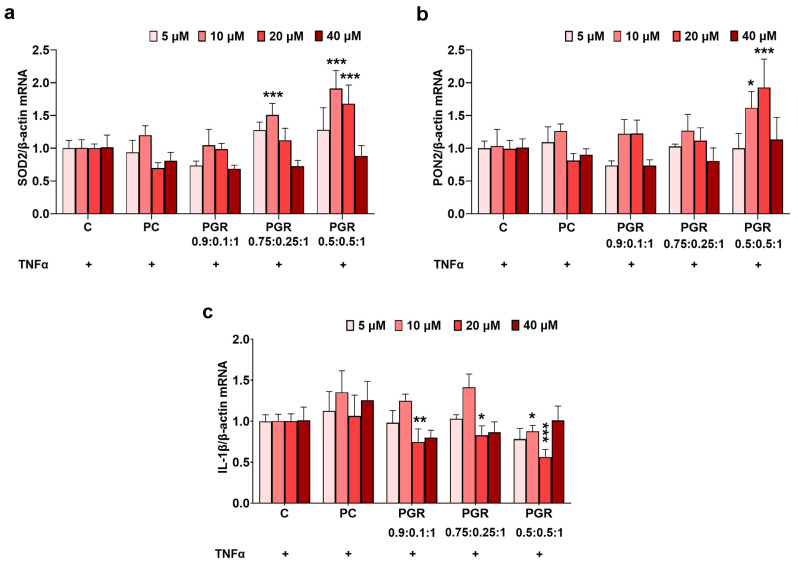
The antioxidant and anti-inflammatory effects of PHYTOGINROSA (PGR) with different mass ratios of GINex:ROSAex:FC (0.9:0.1:1, 0.75:0.25:1 and 0.5:0.5:1) and at different concentrations of 6-gingerol equivalent (5, 10, 20 and 40 µM) in Caco-2 enterocytes activated with TNFα. The gene expression for superoxide dismutase 2 (SOD2) (**a**), paraoxonase 2 (PON2) (**b**) and interleukin 1β (IL-1β) (**c**) are represented relative to that of TNFα-exposed cells. The results are presented as means ± SD. * *p* < 0.05, ** *p* < 0.01, *** *p* < 0.001 for TNFα+PGR vs. TNFα.

The enterocytes form the cellular inner layer of the small intestine, which is the body’s gateway for dietary nutrients and orally administered supplements and drugs. Thus, the enterocytes are the first cells of the small intestine that interact with the active principles of PGR in vivo and mediate the transport of molecules between intestinal lumen and systemic circulation. The obtained results highlight the fact that the PGR 0.5:0.5:1 formulation with a concentration of 10 and 20 µM of 6-gingerol equivalent best stimulated the gene expression of the antioxidant enzymes SOD2 and PON2, and the concentration of 20 µM had the highest inhibition effect on the gene expression of the proinflammatory molecule IL-1β, as shown in Figure 4.

All formulations of PGR showed a significant increasing trend for the SOD2 and PON2 gene expressions, while the IL-1β gene expression had a significant decreasing trend correlated with the increasing 6-gingerol concentration (all *p* < 0.001 according to the one-way ANOVA test). However, the post hoc analysis of these data, improved by the T-test, added important differences between the PGR formulations. Thus, the gene expression of the antioxidant enzyme SOD2 was significantly increased in Caco-2 cells exposed to PGR 0.75:0.25:1 (10 µM: 50%, *p* < 0.001) but to a lesser extent compared with PGR 0.5:0.5:1 (10 µM: 91%, *p* < 0.001; 20 µM: 68%, *p* < 0.001). PGR 0.9:0.1:1 had no antioxidant effect, suggesting that ROSAex in higher concentrations is beneficial because it supports better the antioxidant properties of GINex. The gene expression of the antioxidant enzyme PON2 was significantly increased in Caco-2 cells exposed to PGR 0.5:0.5:1 (10 µM: 56%, *p* < 0.05; 20 µM: 94%, *p* < 0.001). The gene expression of the proinflammatory molecule IL-1β decreased the most in Caco-2 cells incubated with PGR 0.5:0.5:1 (20 µM: 43%, *p* < 0.001) and much less in cells incubated with PGR 0.75:0.25:1 (20 µM: 17%, *p* < 0.05) and PGR 0.9:0.1:1 (20 µM: 25%, *p* < 0.01), (Figure 4). Based on these results, we further used for in vivo experiments PGR in a mass ratio of 0.5:0.5:1.

### 3.4. Evaluation of the PGR 0.5:0.5:1 Cytotoxicity 

**Figure 5:**
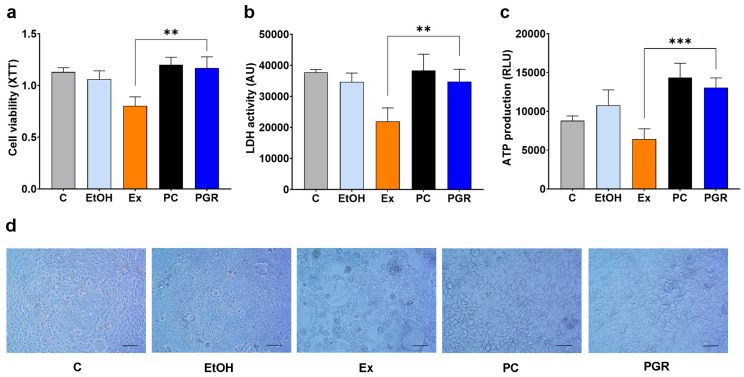
Viability of Caco-2 enterocytes after incubation with PHYTOGINROSA (PGR) compared to phosphatidylcholine (PC), hydroalcoholic extracts (Ex) or ethanol (EtOH). Cell viability (XTT) (**a**), LDH activity (**b**) and intracellular ATPs level (**c**). The results are presented as means ± SD. ** *p* < 0.01, *** *p* < 0.001 for PGR versus Ex. Light microscopy images depicting the cellular density of Caco-2 cells after exposure to control media (C), EtOH, Ex, PC or PGR (**d**); scale bar—50 μm.

The obtained results show that, unlike the hydroalcoholic extracts which are cytotoxic, PGR in the formulation 0.5:0.5:1 and 20 µM equivalent 6-gingerol does not affect the viability of enterocytes measured by the XTT method (a 45% increased viability compared to that of the extracts, *p* < 0.01) or LDH activity (58% increase compared to extracts, *p* < 0.01) (Figure 5a,b). The exposure of cells to PGR determined the increase in intracellular ATP levels as compared to cells exposed to extracts (2 fold, *p* < 0.001), which inhibited the production of ATP (Figure 5c).

### 3.5. Biodistribution of 6-Gingerol from PGR 0.5:0.5:1 in the Plasma and Organs of Mice

**Figure 6:**
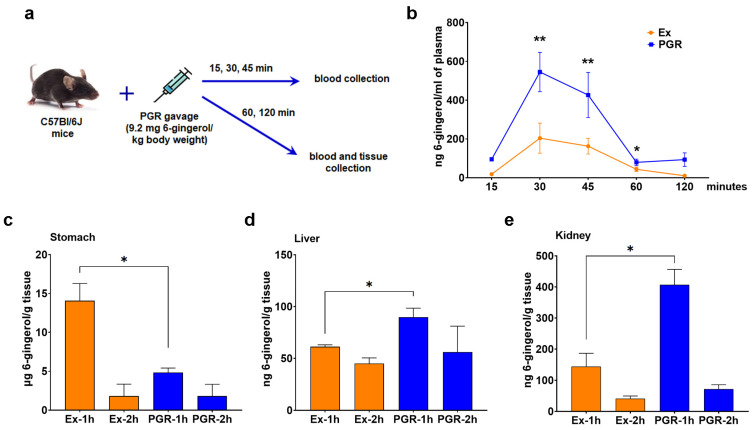
Experimental design (**a**) for the assessment of the bioavailability of free 6-gingerol levels in the plasma (**b**), stomach (**c**), liver (**d**) and kidney (**e**) of C57Bl/6J mice who received PHYTOGINROSA (PGR) compared to those who received extracts (Ex) at 15, 30, 45, 60 and 120 min post-administration. The results are presented as means ± SD for (**b**) and means ± SEM for (**c**–**e**); * *p* < 0.05 and ** *p* < 0.01 for PGR versus Ex.

Free 6-gingerol was detected in the plasma of mice receiving PGR or extracts at a concentration of 545 ng/mL and 204 ng/mL, respectively, 30 min after the gavage. Free 6-gingerol was still detectable 2 h post-administration; the levels were increased in the plasma of PGR mice compared to those receiving extracts (by 2.6 fold, *p* < 0.01 at 30 and 45 min and by 83%, *p* < 0.05 at 60 min) (Figure 6b). 

The level of free 6-gingerol was 65% lower (*p* < 0.05) in the stomach of mice 1 h after administration of PGR compared to those who received the mix of extracts (Figure 6c), suggesting a better absorption of PGR in the digestive tube. In agreement with these results, mice who received PGR presented a 46% (*p* < 0.05) increase in free 6-gingerol levels in the liver and a 2.8-fold (*p* < 0.05) increase in the kidney compared to those that received extracts (Figure 6d,e). The levels of free 6-gingerol in the spleen, pancreas and heart were under the detection limit of the method used.

### 3.6. Assessment of the Antioxidant Effects of PGR 0.5:0.5:1 In Vivo

**Figure 7:**
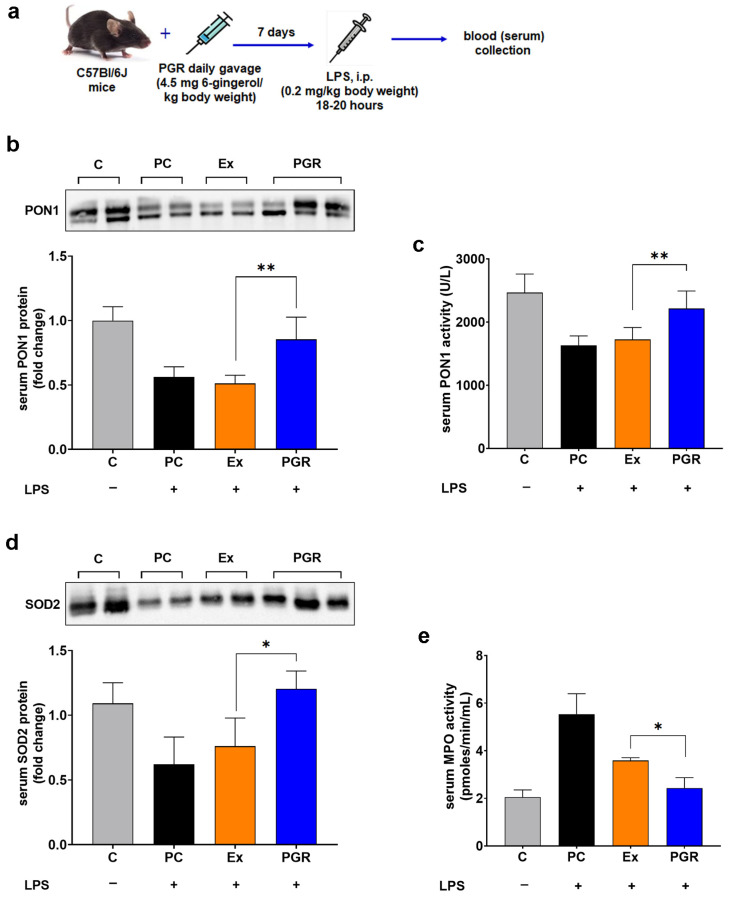
Experimental design for the assessment of the antioxidant effects of PGR 0.5:0.5:1 in vivo (**a**). Levels of the antioxidant enzyme paraoxonase 1 (PON1) protein (**b**) and activity (**c**), superoxide dismutase 2 (SOD2) protein (**d**) and pro-oxidant enzyme myeloperoxidase (MPO) activity (**e**) in the sera of mice who received PHYTOGINROSA (PGR), a mix of extracts (Ex) and phosphatidylcholine (PC) before the induction of systemic inflammation. The results are presented as means ± SD; * *p* < 0.05, ** *p* < 0.01 for PGR versus Ex; C—control animals.

To evaluate the antioxidant effects of PGR in vivo, the levels of the antioxidant enzymes PON1 and SOD2 and the activity of the pro-oxidant enzyme MPO were evaluated in the sera of mice with LPS-induced systemic inflammation who received PGR, extracts or PC prior to LPS injection. Thus, the protein levels of PON1 and SOD2 were over 50% higher in mice treated with PGR compared to those receiving unformulated extracts (67%, *p* < 0.01 for PON1 and 58%, *p* < 0.05 for SOD2) (Figure 7b,d). The PON1 activity was increased by 28%, (*p* < 0.01), reaching the level of the control animals (Figure 7c), while the MPO activity was reduced by 32% (*p* < 0.05) in PGR mice compared to animals treated with extracts (Figure 7e).

### 3.7. Assessment of the Anti-Inflammatory Effects of PGR 0.5:0.5:1 In Vivo

**Figure 8:**
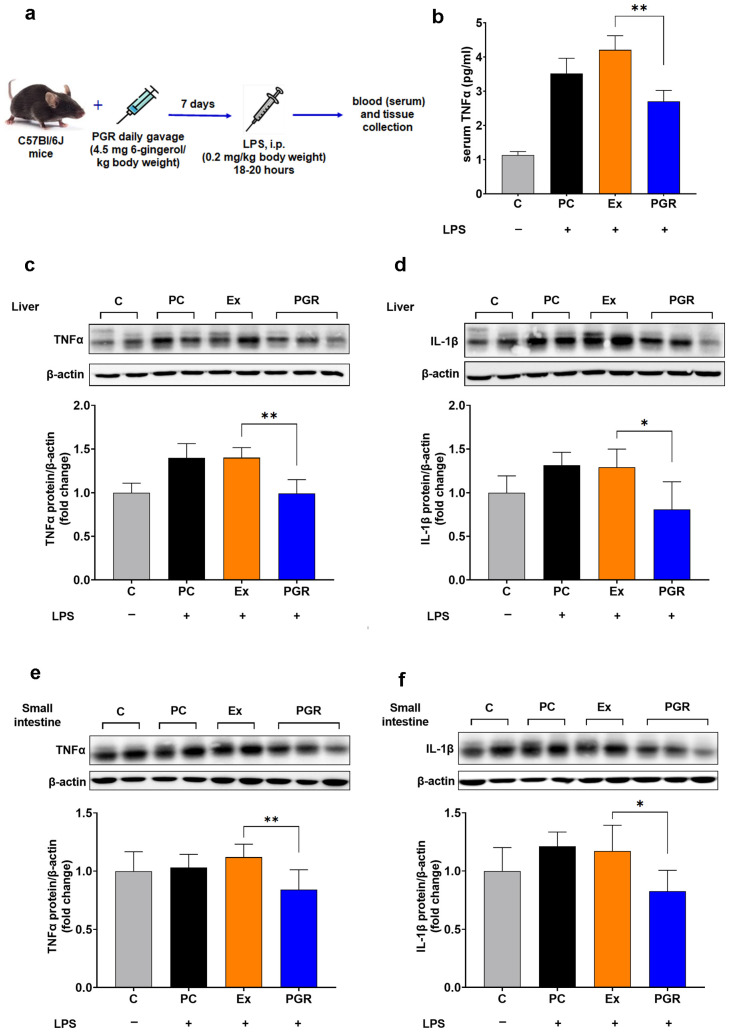
Experimental design for the assessment of the anti-inflammatory effects of PGR 0.5:0.5:1 in vivo (**a**). Levels of tumor necrosis factor α (TNFα) in sera (**b**) and TNFα and interleukin 1β (IL-1β) in the livers (**c**,**d**) and small intestines (**e**,**f**) of mice that received PHYTOGINROSA (PGR), extracts (Ex) and phosphatidylcholine (PC) before LPS-induced systemic inflammation. The results are presented as means ± SD; * *p* < 0.05, ** *p* < 0.01 for PGR versus Ex; C—control animals.

To evaluate the anti-inflammatory potential of PGR, the levels of TNFα were measured in the sera, livers and small intestines of mice with systemic inflammation who, prior to LPS injection, received PGR, extracts or PC. The results show that the administration of PGR determined a 36% reduction in the plasma levels of TNFα compared with mice who received unformulated extracts (*p* < 0.01) (Figure 8b). The levels of the inflammatory markers TNFα and IL-1β in the livers of mice pretreated with PGR were comparable to those in the control animals and significantly decreased compared to mice who received extracts or PC (30%, *p* < 0.01 for TNFα, 37%, *p* < 0.05 for IL-1β) (Figure 8c,d). Similarly, the protein expressions of TNFα and IL-1β in the small intestines of mice pretreated with PGR were significantly decreased compared to mice who received extracts (25%, *p* < 0.01 for TNFα, 30%, *p* < 0.05 for IL-1β) (Figure 8e,f).

### 3.8. Evaluation of the PGR 0.5:0.5:1 Toxicity In Vivo

**Figure 9:**
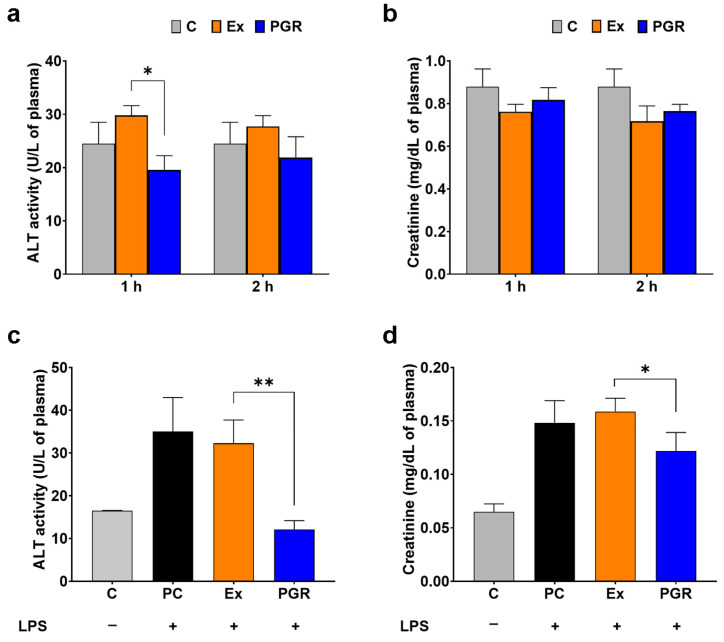
The plasma alanine aminotransferase (ALT) activity (**a**) and creatinine levels (**b**) in mice one hour (1 h) and two hours (2 h) after receiving PHYTOGINROSA (PGR) or extracts (Ex) at a dose of 9.2 mg of 6-gingerol/kg body weight. The sera ALT activity (**c**) and creatinine levels (**d**) in mice who received a dose of 4.5 mg of 6-gingerol/kg body weight PGR, Ex or phosphatidylcholine (PC) prior to LPS-induced inflammation. The results are presented as means ± SD; * *p* < 0.05, ** *p* < 0.01 for PGR compared to Ex; C—control animals.

A single dose of PGR 0.5:0.5:1 equivalent to 9.2 mg of 6-gingerol/kg body weight (from the biodistribution study) induced no increase in ALT activity or creatinine levels in the mouse plasma one and two hours after the gavage (Figure 9a,b). On the contrary, plasma ALT activity was significantly decreased by PGR 0.5:0.5:1 one hour after the gavage compared to extract administration (by 34%, *p* < 0.05) (Figure 9a).

The results show that ALT activity is statistically significantly increased after LPS injection in the sera of mice who received by gavage PC or extracts (by 2-fold, *p* < 0.05). A dose of PGR 0.5:0.5:1 equivalent to 4.5 mg of 6-gingerol/kg body weight administrated daily for seven days before LPS injection dramatically decreased the activity of ALT (62%, *p* < 0.01), maintaining it at a comparable level to the control group (Figure 9c). Similarly, creatinine levels were highly increased by LPS administration (2.5-fold, *p* < 0.01) in the mice that received PC or extracts compared to control mice. PGR administration determined a significant decrease in creatinine levels in the plasma of mice (23%, *p* < 0.05) as compared to unformulated extracts (Figure 9d).

## 4. Discussion

Numerous natural compounds are known to influence oxidative and inflammatory stress. The molecular mechanisms of their action were described and recommended them as remedies complementary to allopathic drugs in different pathologies [47]. Unfortunately, the poor water solubility of natural compounds reduces their bioavailability and limits their therapeutic use. In addition, the intake of biologically active compounds in various formulations, such as powder capsules, alcoholic extracts or encapsulated in nanocarriers, has produced contradictory results. Thus, the need to create novel delivery systems for such natural bioactive compounds, ensuring their increased bioavailability and beneficial effects, led us to develop a new formulation of active compounds from hydroalcoholic extracts of ginger rhizomes and rosehips.

In the presented study, we established for the first time a procedure for the preparation of phytosomes containing the active principles from freeze-dried GINex and ROSAex, providing a stable formulation which ensures an improved bioavailability and biodistribution of the active compounds and consequently increases their antioxidant and anti-inflammatory action.

Ginger and mulberry hydroalcoholic extracts have been previously formulated in phytosomes and proved to exert antimetabolic syndrome and neuroprotective effects in rats subjected to a high-carbohydrate/high-fat diet and cerebral ischemia [48]. Recently, a pilot study evidenced, in patients with knee osteoarthritis, that a lecithin formulation of standardized extracts of *Zingiber officinale* and *Acmella oleracea* is effective in alleviating pain and inflammation, exerting no side effects [19,48,49]. Very recently, PEGylated nanophytosomes containing 6-gingerol were prepared and demonstrated accelerating effects on the wound healing process [50]. Rosehip fruits have been described as having a very complex composition, and various beneficial effects have been suggested for their formulation as powders or extracts [22,24,51]. The differences in the content of these compounds depend on many factors: genetic variation, maturity of the fruit, degree of ripening, growth stage, climate and storage conditions as well as the extraction and analytical method [41]. Unlike ginger extracts, rosehip extracts have not been formulated into phytosomes until now.

Our data evidence for the first time that freeze-dried GINex and ROSAex can be formulated collectively in PC-based phytosomes in various mass ratios. Physical and chemical characterizations of the PGR phytosomes indicate several populations of particles with a good encapsulation efficiency of 6-gingerol and β-carotene and a relatively good stability; their size increases as the concentration of ROSAex increases. The mass ratio that allowed the maximum loading of ROSAex in a relatively stable formulation is GINex:ROSAex:PC—0.5:0.5:1. The stability and solubility of PGR 0.5:0.5:1 are confirmed by the 4-fold increase in the bioaccessibility of 6-gingerol compared to the extract in the conditions that mimic those of the gastrointestinal tract, and these findings are in agreement with previous reports showing a low bioaccessibility of free phenolic compounds after in vitro digestion as compared to encapsulated polyphenols [45,52]. Structural characterization of PGR based on the ^31^P NMR spectra reveals that the shielding of the phosphorus atom in PC is more pronounced as the negative charge from the oxygen atom bound to the phosphorus becomes more localized [53]. The chemical shift of −1.05 measured in PG is in good agreement with the results previously reported [46]. Our data evidence that the shielding effect is proportional to the amount of ROSAex in the PGR. Thus, the PGR with a high content of ROSAex achieved a more efficient isolation of the P-O^−^ group, indicating a more efficient inclusion of PC in the PGR. The PGR with a lower content of ROSAex allowed for a greater exposure of the P-O^−^ group and implicitly nonparticipating electrons to more electropositive groups in neighboring molecules. This can be explained by the stronger binding of the trimethylammonium group (Me_3_N^+^) in PGR proportional with the amount of ROSAex, which leaves the phosphate group (P-O^−^) more shielded from nonparticipating electrons.

Among the three types of PGR tested (mass ratios 0.9:0.1:1, 0.75:0.25:1 and 0.5:0.5:1), the PGR 0.5:0.5:1 formulation at a concentration of 20 µM equivalent 6-gingerol proved to have the most effective antioxidant and anti-inflammatory effects in vitro. These effects are expressed as the PGR’s capacity to induce the gene expressions of SOD2 and PON2 and to reduce the gene expression of IL-1β in TNFα-activated Caco-2 enterocytes. These data suggest that a higher concentration of ROSAex is beneficial because it supports the antioxidant properties of GINex, most likely based on the enriched content of carotenoids. In addition, the PGR 0.5:0.5:1 formulation is protective for enterocytes since it does not affect cell viability or their energetic status, unlike extracts which do exert cytotoxic effects. 

One of the challenges of our endeavor was to formulate GINex and ROSAex in stable phytosomes to ensure an increased bioavailability and biodistribution of the active principles of the extracts. Our results regarding the bioavailability show more than double the concentration of the free 6-gingerol in the plasma of animals receiving the PGR 0.5:0.5:1 compared to those who received the mix of extracts in equivalent concentrations in the interval of 15–120 min. These results are in accordance with those obtained in vitro for the bioaccesibility of 6-gingerol and demonstrate that the formulation of active principles from GINex and ROSAex into phytosomes ensures their increased solubility. The PGR 0.5:0.5:1 presents a better absorption in the digestive tract compared to the mix of extracts, the free 6-gingerol being rapidly accumulated in the livers and kidneys of mice. The amount of free 6-gingerol in the tissue samples could be due to the glucuronide conjugates of gingerols, which are pharmacologically inactive metabolites and are formed in the stomach, enter the systemic circulation and accumulate in various tissues. Once in the tissues, the conjugates are deconjugated to release the free active forms via the action of the β-glucuronidase enzyme, which is expressed in many organs [54,55].

The antioxidant and anti-inflammatory properties have been described over time for both GINex and the ROSAex [8,56,57]. An important achievement of our study was the demonstration of the increased bioavailability and biodistribution of the active compounds from GINex and ROSAex formulated in PGR that were accompanied by a significant improvement in their antioxidant and anti-inflammatory effects in vivo. Our data demonstrate that PGR administration to mice before LPS systemic induction of inflammation generates a better antioxidant and anti-inflammatory defense compared to animals that received the equivalent mix of extracts or PC. Thus, plasma levels of the antioxidant enzymes PON1 and SOD2 were significantly increased, while the activity of the pro-oxidant enzyme MPO was significantly decreased in mice treated with PGR versus extracts. Regarding the anti-inflammatory effects of PGR, the protein expression of TNFα and IL-1β were considerably reduced in the livers and the small intestines of the mice who received PGR compared to those treated with extracts or PC.

## 5. Conclusion

All these data taken together prove that the method described here for the preparation of phytosomes containing GINex and ROSAex allows for the achievement of an improved formula of bioactive compounds extracted from ginger and rosehips, characterized by a greater bioavailability and more powerful antioxidant and anti-inflammatory effects. Our results are all the more important as they refer to the use of biologically active compounds from extracts that have been reported in previous studies to be more efficient compared to isolated purified components and our and others’ data supporting the synergistic action of plants’ active compounds [47]. Moreover, the natural active compounds present the advantage that they have lower adverse effects compared to synthetic drugs, and thus they can be used safely for a longer period of time. 

## Figures and Tables

**Table 1:** Mass ratios of ginger (GINex) and rosehip (ROSAex) extracts and phosphatidylcholine (PC) for PHYTOGINROSA (PGR) and PHYTOGIN (PG) preparation.

Phytosomes (Mass Ratio)	GINex (mg)	ROSAex (mg)	PC (mg)	EtOH (mL)	Temperature (°C)	Reflux Time (h)
PGR 0.5:0.5:1	500	500	1000	125	60	2
PGR 0.75:0.25:1	750	250	1000	125	60	2
PGR 0.9:0.1:1	900	100	1000	125	60	2
PG 1:1	1000	-	1000	125	60	2

**Table 2:** The content of total polyphenols, flavonoids, 6-gingerol and 6-shogaol in freeze-dried GINex (grams/100 grams GINex).

Bioactive Compounds	Grams/100 grams GINex
Total polyphenols (eq. gallic acid)	40.88 ± 0.48
Total flavonoids (eq. quercetin)	1.70 ± 0.17
6-gingerol (UHPLC)	3.00 ± 0.38
6-shogaol (UHPLC)	0.29 ± 0.01
Total gingerols (UHPLC)	9.00 ± 0.09

**Table 3:** The content of total polyphenols, total flavonoids, β-carotene and total carotenoids in freeze-dried ROSAex (grams/100 grams).

Bioactive Compounds	Grams/100 grams ROSAex
Total polyphenols	5.42 ± 0.09
Total flavonoids	0.041 ± 0.005
Total carotenoids (UHPLC)	0.342 ± 0.031
β-carotene (UHPLC)	0.297 ± 0.092

**Table 4:** The values of the Z-average, polydispersity index (PDI) and Zeta potential of phosphatidylcholine (PC), PHYTOGIN (PG) and PHYTOGINROSA (PGR) in the three formulations.

Sample	Z-Average (nm)	PDI	Zeta Potential (mV)
PC	103.78 ± 6.39	0.32 ± 0.02	−19.02 ± 1.34
PG	119.95 ± 8.64	0.39 ± 0.02	−22.66 ± 2.44
PGR 0.9:0.1:1	193.72 ± 39.48	0.42 ± 0.05	−18.17 ± 1.85
PGR 0.75:0.25:1	335.78 ± 61.18	0.56 ± 0.06	−21.10 ± 0.84
PGR 0.5:0.5:1	783.14 ± 219.26	0.50 ± 0.09	−18.90 ± 1.48

**Table 5:** Drug loading and encapsulation efficiency (%) in PHYTOGINROSA (PGR) and PHYTOGIN (PG) phytosomes.

Grams/100 grams	PGR 0.5:0.5:1	EE%	PGR 0.75:0.25:1	EE%	PGR 0.9:0.1:1	EE%	PG 1:1	EE%
6-gingerol	0.500 ± 0.120	83.3 ± 3.4	0.844 ± 0.101	89.2 ± 2.7	1.020 ± 0.021	86.3 ± 3.1	1.14 ± 0.13	91.3 ± 3.0
β-carotene	0.074 ± 0.009	94.3 ± 4.5	0.030 ± 0.002	91.3 ± 3.1	0.010 ± 0.001	85.4 ± 2.1	-	-

**Table 6:** Residual drug loading of PHYTOGINROSA (PGR) 0.5:0.5:1 compared to ginger extract (GINex) for 1 year of storage.

Time at 4 °C (months)	Freeze-Dried GINex (6-Gingerol,%)	Freeze-Dried PGR (6-Gingerol,%)
0	3.00 ± 0.38	0.500 ± 0.120
1	2.82 ± 0.25	0.521 ± 0.098
3	2.67 ± 0.18	0.517 ± 0.081
6	2.37 ± 0.25	0.508 ± 0.046
12	2.19 ± 0.18	0.509 ± 0.058

## Data Availability

Data are available from the corresponding author upon request.

## Supplementary Materials

The following supporting information can be downloaded at: https://www.mdpi.com/article/10.3390/pharmaceutics15041066/s1, Table S1: Sequences of primers for human genes; Figure S1: UHPLC–DAD chromatograms showing 6-gingerol and 6-shogaol; Figure S2: UHPLC–DAD chromatograms showing β-carotene.
