# Supplementary material for: Formulation of Phytosomes with Extracts of Ginger Rhizomes and Rosehips with Improved Bioavailability, Antioxidant and Anti-Inflammatory Effects In Vivo
Source: Pharmaceutics. 2023 Mar 25;15(4):1066. doi: 10.3390/pharmaceutics15041066 (PMC10146199; doi:10.3390/pharmaceutics15041066)
Supplement: Supplementary file 1 [file pharmaceutics-15-01066-s001.zip › pharmaceutics-2244420-SI.pdf]

Title:

Formulation of phytosomes with extracts of ginger rhizomes and rosehips with improved bioavailability, antioxidant and anti-inflammatory effects in vivo

for the Special Issue: "**The Study of Plant Compounds in Antioxidant Activity and Anticancer Activity"**

Authors:

Mariana Deleanu[1], Laura Toma[1], Gabriela Maria Sanda[1], Teodora Barbălată[1], Loredan Ştefan Niculescu[1], Anca Volumnia Sima[1], Calin Deleanu[2,3], Liviu Săcărescu[3], Alexandru Suciu[4], Georgeta Alexandru[4], Iuliana Crişan[4], Mariana Popescu[4], Camelia Sorina Stancu[1*]

Affiliations:

[1]Institute of Cellular Biology and Pathology "Nicolae Simionescu" of the Romanian Academy, 8 B.P. Haşdeu street, Bucharest, Romania

[2] "Costin D. Nenitescu" Institute of Organic and Supramolecular Chemistry of the Romanian Academy, 202B Splaiul Independenței street, Bucharest, Romania

[3] "Petru Poni" Institute of Macromolecular Chemistry of the Romanian Academy, Aleea Grigore Ghica Voda 41A, Iasi, Romania

[4]Hofigal Export Import S.A.,2 Intrarea Serelor, Bucharest, Romania

***Corresponding author:**

Camelia Sorina Stancu

Institute of Cellular Biology and Pathology "Nicolae Simionescu" of the Romanian Academy

8, B.P.Hasdeu Street, 050568 Bucharest, Romania

Phone: +4021 319 4518

Fax: +4021 319 4519

E-mail: camelia.stancu@icbp.ro

2. Material and Methods

2.2. Preparation and characterization of ginger extracts

UHPLC-DAD analysis of gingerols and shogaols in hydroalcoholic extract of ginger (GINex) and PHYTOGINROSA

Chromatographic separation of the GINex compounds was performed using an ultrahigh performance liquid chromatography (UHPLC) equipped with a binary pump, vacuum degasser, column oven, temperature-controlled autosampler and diode array detector (DAD) (Agilent Technologies 1290 Infinity). A Zorbax SB-C18 column (2.1x100 mm, 1.8 µm, Agilent, USA) was used. The mobile phase was composed by:0.1% formic acid (A) and 0.1% formic acid acetonitrile (B). Separation of compounds was carried out with a gradient elution profile of the solvent B: 0 min - 40%, 1 min - 40%, 9 min - 80%, 10 min - 95%, 13 min - 95%, 15 min - 40%, 16 min - 40%, with a 0.3 ml/min flow rate. The detection was done at λ=282 nm, and column temperature 50°C. The standard curves of 6-gingerol and 6-shogaol were constructed in the range 5-50 µg/ml and 5µL were injected into UHPLC system. The results were expressed as g/100g GINex of 6-gingerol and 6-shogaol [1].

Determination of total phenolic content

The total amount of phenolic compounds (TP) of GIN was determined by the Folin–Ciocâlteu method (Nagendra chari et al., 2013) with some modifications: 200 µL of diluted sample in ethanol/standard solutions (gallic acid) were mixed with 1.0 ml diluted Folin – Ciocâlteu phenol reagents (1:10). After 4 min, 0.8 ml of 7.5% Na2CO3 in water was added to the mixture. After 2 h incubation in darkness, the samples were centrifuged and absorbance was measured at 765 nm using a microplate reader (Infinite M200 PRO, TECAN) against blank sample. The concentration of TP was assessed using gallic acid in range 5–500 µg/ml for calibration curve. The results were expressed as g/100g GINex (gallic acid eq.) [1].

Determination of total flavonoids

The total flavonoids content (TF) in GIN was determined according to the Dowd method (Adel and Prakash, 2010). Briefly, 200 µl of 2% AlCl3 in 80% ethanol was mixed with the same volume of the diluted sample in ethanol. After 10 min, the absorbance was measured at 430 nm against a blank sample consisting of extract solution with 200 µl of ethanol without AlCl3. The TF content was determined using a standard curve with quercetin in the range 5–100 µg/ml and expressed as g/100g GINex (quercetin eq.) [1].

2.3. Preparation and characterization of rosehip extract

Determination of β-caroten and total carotenoids of rosehip hydroalcoholic extract (ROSAex) and PHYTOGINROSA

The content in total carotenoids was determined by a UHPLC method [2] with some modifications. For the extraction of carotenoids an extraction mixture composed of hexane/acetone/ethanol in a 50/25/25 ratio was used. Thus, the extraction mixture was added to the freeze-dried ROSAex (accurately weighted) and sonicated on the water bath for 20 minutes. The mixture was then centrifuged at 10,000xg for 10 minutes. The organic layer was transferred to another tube and repeated the extraction in the same conditions. The combined organic phases were evaporated to dryness under a stream of nitrogen. The residue was dissolved in a mixture of tetrahydrofuran/acetonitrile/methanol in a ratio of 15/30/55. The sample vortexed and filtered through a 0.22 µm filter was then injected into the UHPLC system (Agilent Technologies 1290 Infinity).

The separation of carotenoids, was carried out on a Zorbax SB C18 column, 2.1 x 100 mm, 1.8 µm, with a mobile phase composed of A - methanol 90% and B - acetonitrile 10%, at a flow rate of 0.3 mL/min, in isocratic mode, for 50 minutes. The temperature in the column was 30°C, and the detection was done at λ = 450 nm. The standard curves for β-carotene was constructed in the range 1-40 µg/ml and 5µL were injected into UHPLC system. The results were expressed as g/100g ROSAex of β-carotene.

Table S1. Sequences of primers for human genes whose expression was assessed by quantitative Real-Time PCR

Gene	Ref Seq	Forward	Reverse	Amplicon (bp)
**SOD2**	NM_000636.3	TTTCAATAAGGAACGGGGACAC	GTGCTCCCACACATCAATCC	109
**PON2**	NM_000305.2	TCTGGCACTCAGAAATCGACT	CAGACCATTGGGAAGTATGTCAA	124
**IL-1β**	NM_000576.2	ATGATGGCTTATTACAGTGGCAA	GTCGGAGATTCGTAGCTGGA	132
**β-actin**	NM_001101.3	GTCTTCCCCTCCATCGT	CGTCGCCCACATAGGAAT	82

Results

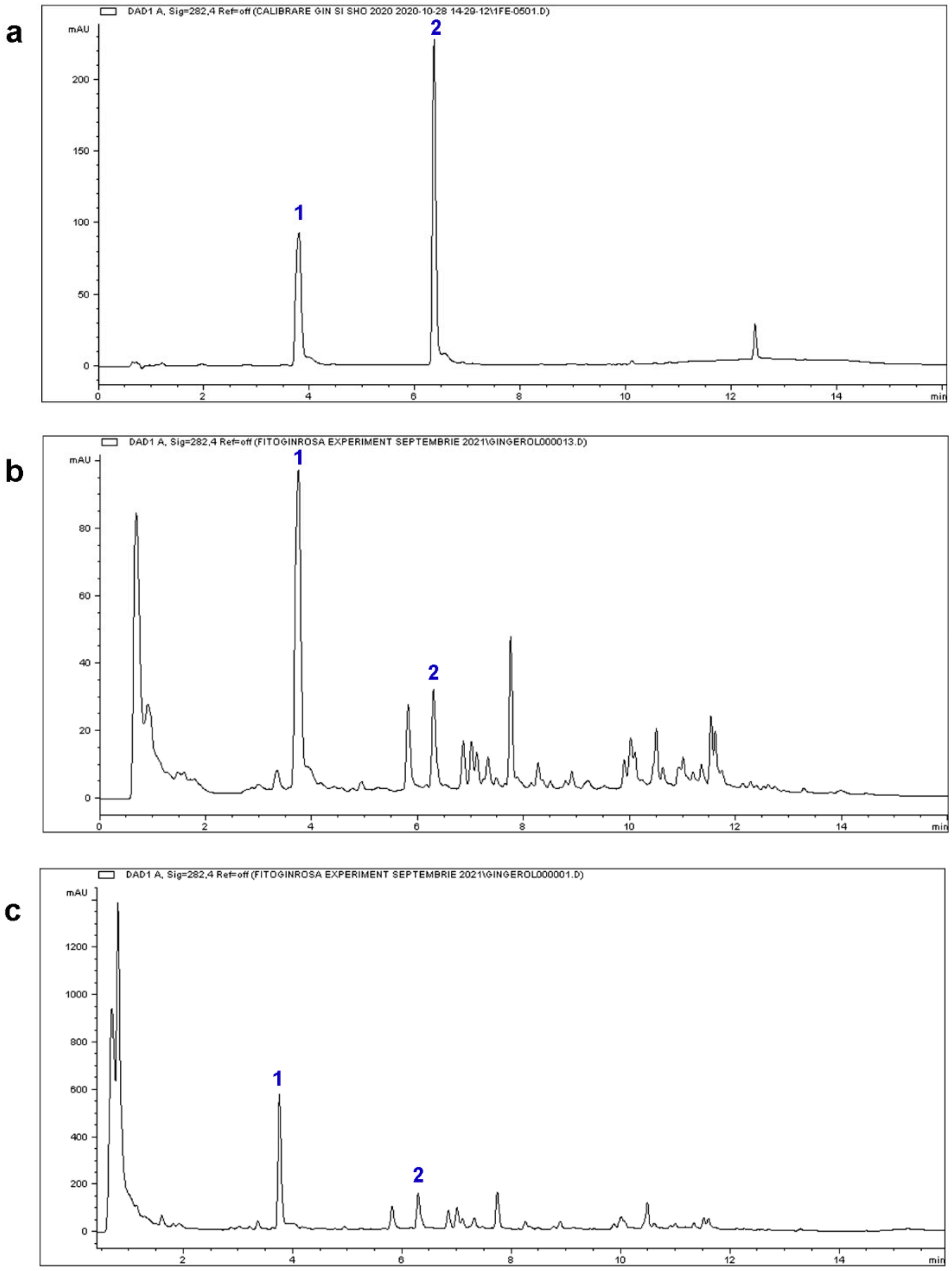

Figure S1. UHPLC–DAD chromatograms showing the 6-gingerol and 6-shogaol standards (a), the hydroalcoholic extract of ginger rhizomes (GINex) (b) and PHYTOGINROSA (PGR) (c) at 282 nm; (1) 6-gingerol and (2) 6-shogaol.

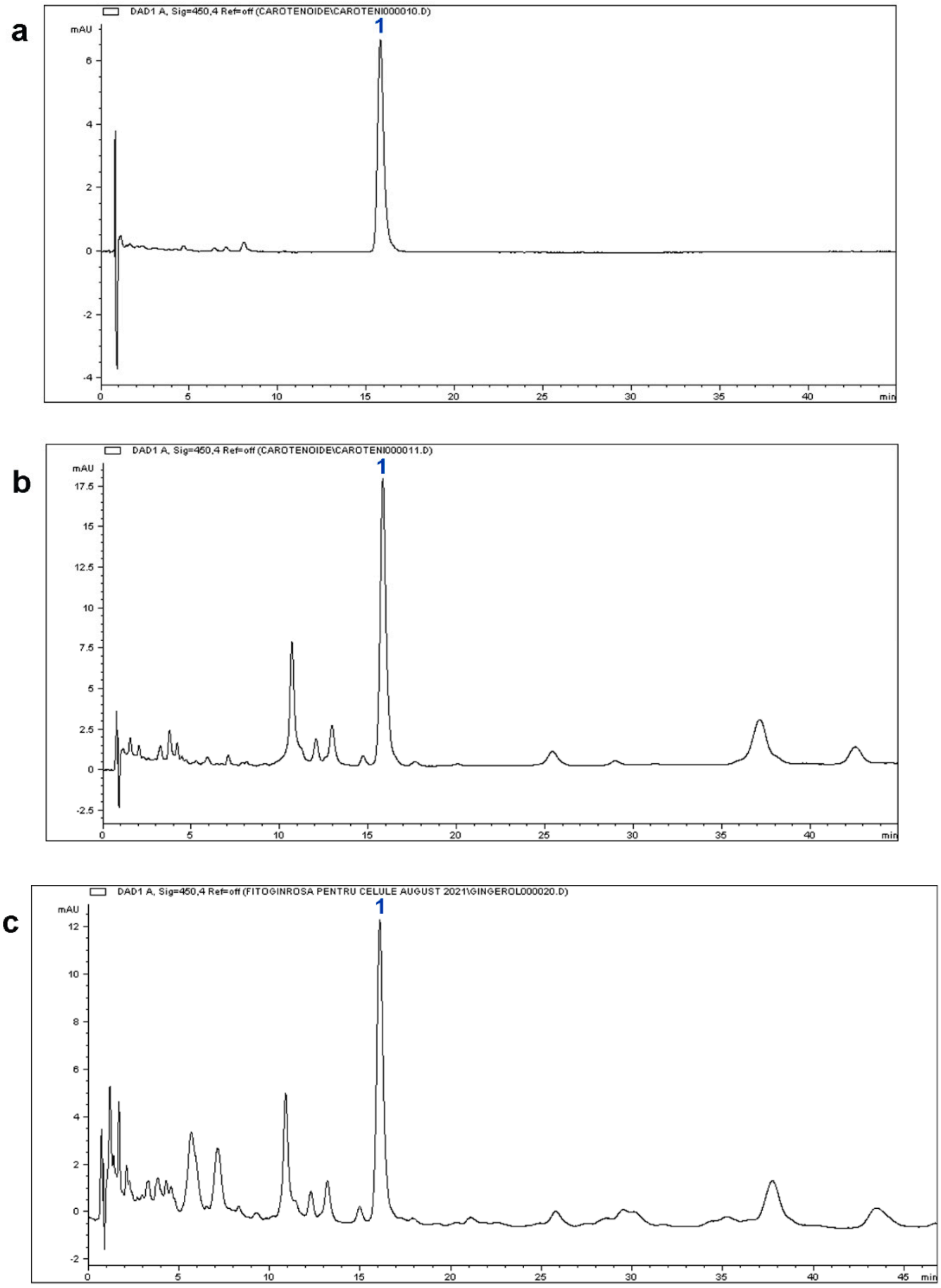

Figure S2. UHPLC–DAD chromatograms showing the β-carotene standard (a), the rosehip extract (ROSAex) (b) and PHYTOGINROSA (PGR) (c) at 450 nm; (1) β-carotene.

References

1. Carnuta, M.G.; Deleanu, M.; Barbalata, T.; Toma, L.; Raileanu, M.; Sima, A.V.; Stancu, C.S. Zingiber officinale extract administration diminishes steroyl-CoA desaturase gene expression and activity in hyperlipidemic hamster liver by reducing the oxidative and endoplasmic reticulum stress. *Phytomedicine* **2018**, *48*, 62-69, doi:10.1016/j.phymed.2018.04.059.
2. Barba, A.I.O.; Hurtado, M.C.; Mata, M.C.S.; Ruiz, V.F.; Tejada, M.L.S.d. Application of a UV–vis detection-HPLC method for a rapid determination of lycopene and β-carotene in vegetables. *Food Chemistry* **2006**, *95*, 328-336, doi:https://doi.org/10.1016/j.foodchem.2005.02.028.
